# Gallic acid regulates immune response in a mouse model of rheumatoid arthritis

**DOI:** 10.1002/iid3.782

**Published:** 2023-02-16

**Authors:** Siqin Liu, Jiajia Li, Le‐heng Feng

**Affiliations:** ^1^ Department of Laboratory Daqing Oilfield General Hospital Daqing Heilongjiang China; ^2^ Department of Rheumatism, The First Hospital of Qiqihar Affiliated Qiqihar Hospital of Southern Medical University Qiqihar Heilongjiang China

**Keywords:** collagen‐induced arthritis, gallic acid, inflammation, Th17, Treg

## Abstract

**Introduction:**

Previous studies revealed that gallic acid (GA) exerts anti‐inflammation and immuno‐regulatory properties. This study aims to explore the pharmacological activities of GA in collagen‐induced arthritis (CIA) mouse model.

**Methods:**

Male DBA/1J mice were used to construct the CIA model. The mice were administrated with GA for 3 weeks. Clinical arthritis scores and hind paw volume were evaluated over the experimental period. qPCR and Western blot analysis were used to determine the levels of matrix metallopeptidases (MMPs) and cytokines. In addition, flow cytometry was used to measure the populations of Th17 and Treg cells. ELISAs were used to determine the cytokines in the serum and ankle joint tissues.

**Results:**

Treatment of GA (40 and 80 mg/kg/d) reduced clinical arthritis scores and hind paw volume in the CIA mouse model. Besides, treatment of GA reduced the overexpression of MMPs and modulated the dysregulation of inflammation‐related cytokines. Flow cytometry showed that treatment of GA decreased the population of Th17 cells, and increased the population of Treg cells, as supported by treatment of GA regulated the Th17/Treg‐related cytokines.

**Conclusions:**

GA attenuates symptoms in the CIA mouse model by anti‐inflammation and regulating Th17/Treg cell imbalance.

## Graphical abstract

1. Treatment of GA (40 and 80 mg/kg/d) reduced clinical arthritis scores and hind paw volume in the CIA mouse model. 2. GA attenuates symptoms in the CIA mouse model by anti‐inflammation and regulating Th17/Treg cell imbalance.

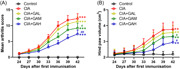

## 1 INTRODUCTION

Rheumatoid arthritis (RA) is known as a progressive autoimmune disease.^1^ It is also known as a chronic inflammatory disease, which is characterized by a series of features including synovial inflammation, bone erosion, and cartilage destruction, as well as the release of inflammatory cytokines and autoantibodies in the circulation.^1^, ^2^ RA is known to be associated with cardiovascular diseases, diabetes, and interstitial lung disease, which causes an increase in morbidity.^3^, ^4^ According to a meta‐analysis, RA affects up to 0.46% of the population from 1995 to 2005, and approximately 460 per 100,000 people suffered from RA worldwide.^5^, ^6^ A series of therapeutic options including disease‐modifying anti‐rheumatic drugs, steroids, and nonsteroidal anti‐inflammatory drugs, and biological response modifiers are recommended based on the severity of RA symptoms and stages.^7^, ^8^ However, well‐documented risks, as well as some side effects, are major limitations of these medications.^7^, ^8^ Therefore, discovering novel agents with good pharmacological and manageable side effects are urgently needed.

Th17/Treg cell imbalance has been implicated to be associated with RA.^9^, ^10^ Th17 and Treg are known as two different subsets of T cells.^11^ Th17 cells trigger an inflammatory response and autoimmunity and Treg cells suppress inflammation and immune response.^9^, ^11^, ^12^, ^13^ A shift in the Th17/Treg cell to the Th17 cell side is associated with an increase in disease activity, whereas the lower ratio of Th17/Treg cell is correlated to the amelioration of RA symptoms. Many studies have supported that patients with RA display a disrupted Th17/Treg balance manifest.^9^, ^14^ For instance, previous studies revealed an increase in the IL‐17 and receptor (RORγt) and a decrease in the populations of Treg cells and its master regulator, forkhead box P3 (FOXP3), in RA patients.^9^ Importantly, recent studies found that targeting Th17/Treg cell imbalance might be an effective strategy for RA therapy, which is supported by a series of preclinical studies.^13^, ^15^ For instance, Du et al. reported that leonurine restores Th17/Treg balance and thereby inhibiting synovial fibroblast action.^16^ Another study reported that treatment of Protectin DX attenuates RA symptoms (e.g., joint injury) and inflammatory responses by restoring Th17/Treg balance.^17^

Gallic acid (GA) is a phenolic acid present in fruits, vegetables, and medicinal herbs and is known for its strong free radical scavenging activities and anti‐inflammatory properties.^18^ Studies have revealed that GA exerts broad pharmacological activities against inflammation‐related diseases including allergic inflammation, inflammatory bowel diseases, pelvic inflammation, and RA.^18^, ^19^ In vitro data suggested that GA triggers the apoptosis of fibroblast‐like synovial cells by promoting the proapoptotic factors (e.g., p53 and Bax) and suppressing the antiapoptotic factors (e.g., Bcl and p‐Akt).^20^ These results suggested that GA might be effective for the treatment of RA. However, it is still unknown the in vivo therapeutic effects of GA on RA. Besides, GA is known as a multi‐target small molecule. It is interesting to explore whether GA's anti‐RA activities are through other signaling pathways or cellular events. Therefore, in collagen‐induced arthritis (CIA) mouse model with or without GA treatment, not only did we evaluate the release of inflammatory cytokines, but also, we explored the changes in Th17/Treg balance.

## 2 MATERIALS AND METHODS

### 2.1 Experimental design and GA administration

Forty male DBA/1 J mice (7‐week‐old) were obtained from GemPharmatech and kept under the specific pathogen‐free condition. The animal studies were approved by the First Hospital of Qiqihar.

The CIA mouse model was constructed as previously described.^21^ In brief, the CIA model was induced by type II collagen combined with Freund's incomplete adjuvant. Type II collagen was dissolved in 0.05 M acetic acid. On Day 1, emulsification was performed by mixing with an equal volume of Freund's complete adjuvant. On Day 21, Freund's incomplete adjuvant alone was injected to boost the immune response. Mice were placed in a restrainer and the tails were sanitized with 75% alcohol swabs. Then the injection was carried out at the site of one third of the way down the tail.

A total of 32 mice with a group of eight mice were assigned. In the GA group, the CIA mice were administrated with GA (20, 40, or 80 mg/kg/d, corresponding to GAL, GAM, and GAH) from Days 21 to 42. GA (purity ≥ 98%) was obtained from Sigma Chemical Co and was dissolved in the phosphate‐buffered saline (PBS) buffer. The doses were selected according to a previous study. In the CIA group, the mice were administrated with PBS daily at the same volume. In addition, another eight DBA/1J mice at the same age were assigned to the control group and administrated with PBS daily.

### 2.2 Clinical arthritis scoring and hind paw volume measurement

Clinical arthritis scoring was determined every 3 days from Days 24 to 42. The clinical arthritis scoring was evaluated based on erythema, swelling, and loss of function. Normal (no signs of arthritis) was scored as 0. Swelling and/or erythema observed in one paw was scored as 1. Swelling and/or erythema observed in both paws was scored as 2. Swelling and/or erythema observed in three and/or four paws was scored as 3. Severe arthritis observed in the entire paw was scored as 4. Besides, a plethysometer (Ugo Basile) was used to determine the volume of two hind paws.

### 2.3 Flow cytometry

Mice were killed 24 h after the last administration of GA. Spleen tissue was collected. A 0.83% ammonium chloride solution was used to prepare a single‐cell suspension. EasySep™ CD4+ T cell isolation kit was obtained from STEMCELL Technologies. Fluorescence dye‐conjugated antibodies used in this study include antibody against CD4‐FITC, antibody against CD25‐APC, antibody against IL‐17A‐APC, and antibody against FoxP3‐PE, which were purchased from eBioScience™ Antibodies (Thermo Fisher Scientific). Single‐cell suspension was prepared and the CD4+ T cells were isolated. Next, the cells were incubated with fluorescence dye‐conjugated antibodies in flow cytometry staining buffer for 30 min in the dark. Flow cytometer was used to analyze the cell suspension and FlowJo was used to analyze the frequencies of each cell population.

### 2.4 Quantitative polymerase chain reaction

Total RNAs were isolated from the ankle joint with TRIzol (Invitrogen). Primers were synthesized by GenScript. The primer sequence was listed in Table 1. cDNA library was constructed by using a reverse transcription reaction. A PCR reaction was then operated and the mRNA expression levels of each target gene were normalized to the β‐actin.

**Table 1:** Oligonucleotide primer sequences for qRT‐PCR.

Gene	Primer direction	Sequence (5′–3′)
MMP3	Forward	GGGAAGCTGGACTCCAACAC
	Reverse	GCGAACCTGGGAAGGTACTG
MMP9	Forward	CACGGTTGGCCCTACAGGCG
	Reverse	AGGCCTCAGAAGAGCCCGCA
MMP13	Forward	GCACTGCTGGGCACCATGCAT
	Reverse	GGGAAGGGGCAGGGACCAACA
IL‐17A	Forward	TCTCCACCGCAATGAAGACC
	Reverse	CACACCCACCAGCATCTTCT
IL‐6	Forward	CACGGCCTTCCCTACTTCAC
	Reverse	CTGCAAGTGCATCATCGTTGT
IL‐10	Forward	AGTGTGTATTGAGTCTGCTGG
	Reverse	TTTCCAAGGAGTTGTTTCCG
TGF‐β	Forward	GCCATGAGCGGTCCATCACG
	Reverse	CAGTCAGCATCCACGCACCAC
GAPDH	Forward	AATGGATTTGGACGCATTGGT
	Reverse	TTTGCACTGGTACGTGTTGAT

Abbreviation: RT‐qPCR, reverse transcription‐quantitative polymerase chain reaction.

### 2.5 Western blot analysis

Protein was isolated from the ankle joint with RIPA buffer and qualified by using the BCA assay. Next, protein samples were loaded into Pre‐cast Gels before membrane transfers. The membranes were incubated with blocking buffer (#P0239, Beyotime) for 1 h. Next, primary antibodies against MMP3 (1:1000), MMP9 (1:1000), MMP13 (1:1000), IL‐6 (1:1500), IL‐10 (1:1200), IL‐17A (1:1000), TGF‐β (1:1200), or β‐actin (1:2000) were added. All antibodies were provided by Abcam. After blotting with primary antibodies overnight at 4°C, the membrane was then incubated with secondary antibodies at room temperature for another 1 h. Normalization was performed by comparing the expressions of each target protein to the β‐actin.

### 2.6 ELISAs

The blood was collected and serum was separated. In addition, ankle joints were collected and tissue lysate was prepared. The levels of MMPs and cytokines in serum and tissue lysate were then determined by using ELISAs, according to the document provided by the manufacturer (R&D Biosystems).

### 2.7 Data analysis

The data were shown as the means ± standard deviation (SD). GraphPad was applied for the data analysis. To analyze the statistical difference, one‐way ANOVA with Tukey's multiple‐comparisons test, or two‐way ANOVA with Bonferroni test was applied. A *p* value that is less than 0.05 indicated statistical difference.

## 3 RESULTS

### 3.1 GA ameliorated symptoms in the CIA mouse model

**Figure 1:**
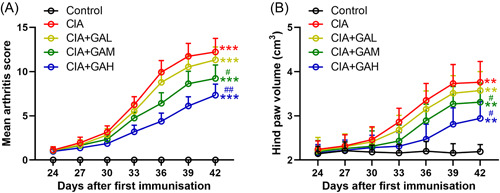
Treatment of gallic acid (GA) ameliorated symptoms in the mouse model of arthritis induced by collagen. After the first emulsification, (A) clinical arthritis scores (A) and hind paw volume (B) were determined from Days 24 to 42. The data were shown as the means ± SD (*n* = 8). **p < .01, ***p < .001 as compared to the control. ^#^ *p* < .05, ^##^ *p* < .01 as compared to the CIA group. GAL, GAM, and GAH corresponded to the GA administration dosage of 20, 40, or 80 mg/kg/d.

First, we determined the effects of GA on the CIA symptoms by using clinical arthritis scores and measuring hind paw volume. As expected, in the CIA group, we observed that the clinical arthritis scores and hind paw volume were significantly increased, indicating the CIA model was successfully established (Figure 1A,B). Treatment of GA (20 mg/kg) did not significantly alter clinical arthritis score and hind paw volume as compared to the CIA group. However, treatment of GA (40 and 80 mg/kg) significantly reduced the clinical arthritis scores and hind paw volume (Figure 1A,B), suggesting that treatment of GA ameliorated symptoms in the CIA mouse model.

### 3.2 GA reduced MMPs’ overexpression in ankle joint in the CIA mouse model

**Figure 2:**
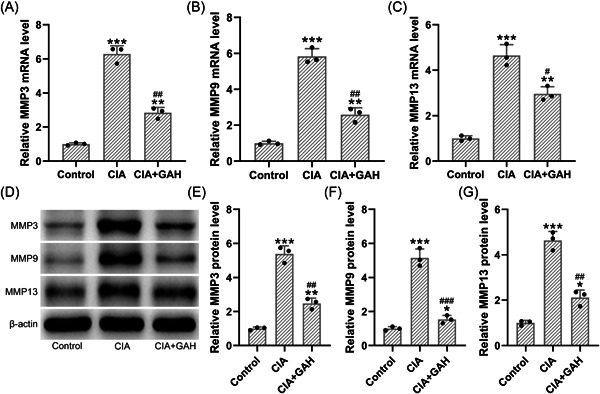
Treatment of GA reduced matrix metallopeptidases overexpression in the ankle joints in the mouse model of arthritis induced by collagen. (A–C) The mRNA levels of MMP3 (A), MMP9 (B), and MMP13 (C) in ankle joint were determined by qPCR. (D) Besides, the protein expressions of MMP3, MMP9, and MMP13 were determined by using Western blot analysis. (E–G) The relative expressions were normalized to the internal control (*n* = 3; Eight tissue homogenates were mixed). *p < .05, **p < .01, and ***p < .001 as compared to the control. ^#^ *p* < .05, ^##^ *p* < .01, ^###^ *p* < .001 as compared to the CIA group. GAH corresponded to the GA administration dosage of 80 mg/kg/d.

Second, we measured the effects of GA on the expressions of MMP. We found that the mRNA levels of MMPs, including MMP3, MMP9, and MMP13, were significantly increased in the CIA group, whereas treatment of GA (80 mg/kg) reduced the mRNA levels of those biomarkers (Figure 2A–C). Consistently, we observed that the protein expressions of MMPs were elevated in the ankle joint after collagen induction. Interestingly, treatment of GA (80 mg/kg) also reduced the protein expressions of those MMP (Figure 2D–G). These results supported that GA reduced MMPs’ overexpression in ankle joints in the mouse model of CIA.

### 3.3 GA‐regulated inflammatory cytokines in the CIA mouse model

**Figure 3:**
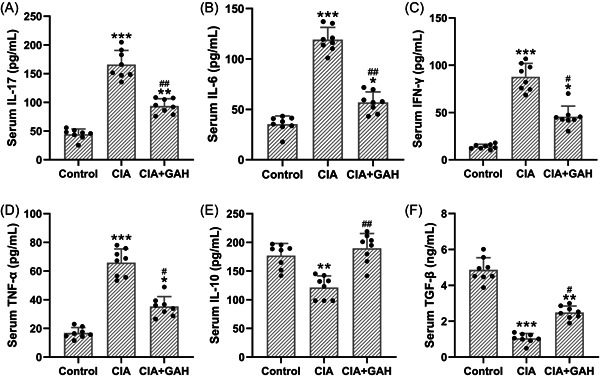
Treatment of GA‐regulated serum inflammation‐related cytokines in the mouse model of arthritis induced by collagen. The levels of IL‐17 (A), IL‐6 (B), IFN‐γ (C), TNF‐α (D), IL‐10 (E), and TGF‐β (F) in serum were measured by specific ELISAs. The data were shown as the means ± SD (n = 8). *p < .05, **p < .01, and ***p < .001 as compared to the control. ^#^ *p* < .05 and ^##^ *p* < .01 as compared to the CIA group. GAH corresponded to the GA administration dosage of 80 mg/kg/d.

Furthermore, we investigated the effects of GA on the inflammation‐related cytokines in the CIA mouse model. We observed that cytokines including IL‐17, IL‐6, IFN‐γ, and TNF‐α were elevated (Figure 3A–D), whilst cytokines including IL‐10 and TGF‐β were reduced in the CIA mouse model (Figure 3E,F), indicating the dysregulation of inflammation in the circulation. However, treatment of GA (80 mg/kg) regulated the expressions of inflammation‐related biomarkers, as suggested by the decrease of IL‐17, IL‐6, IFN‐γ, and TNF‐α and the increase of IL‐10 and TGF‐β in the GA‐treated group.

### 3.4 GA‐regulated Th17/Treg cell balance in the CIA mouse model

**Figure 4:**
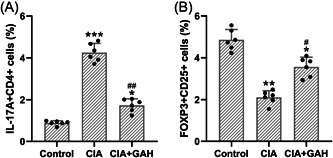
Treatment of GA‐regulated Th17 and Treg cell differentiation balance in the mouse model of arthritis induced by collagen. Splenocytes were isolated (CD4+ T cell) and the frequencies of Th17 (CD4+IL‐17+) and Treg (CD25+FOXP3+, gated on CD4+ cells) were analyzed by using flow cytometry. The graphs represented the percentage of IL‐17A+CD4+ T cells (A) and FOXP3+CD25+ T cells (B). The data were shown as the means ± SD (*n* = 6). *p < .05, **p < .01, and ***p < .001 as compared to the control. ^#^ *p* < .05 and ^##^ *p* < .01 as compared to the CIA group. GAH corresponded to the GA administration dosage of 80 mg/kg/d.

Next, we evaluated the effects of GA on the Th17 and Treg cell differentiation balance. Our results showed an increase of the population of CD4+IL‐17+ T cells and a decrease of the population of CD25+FOXP3+ T cells in the CIA mouse model (Figure 4A). However, treatment of GA (80 mg/kg) decreased the frequencies of CD4+IL‐17+ T cells, and increased the frequencies of CD25+FOXP3+ T cells (Figure 4B), indicating that GA‐regulated Th17 and Treg cell balance in the CIA mouse model.

### 3.5 Treatment of GA‐regulated cytokines relevant to Th17 and Terg cells in the ankle joints

**Figure 5:**
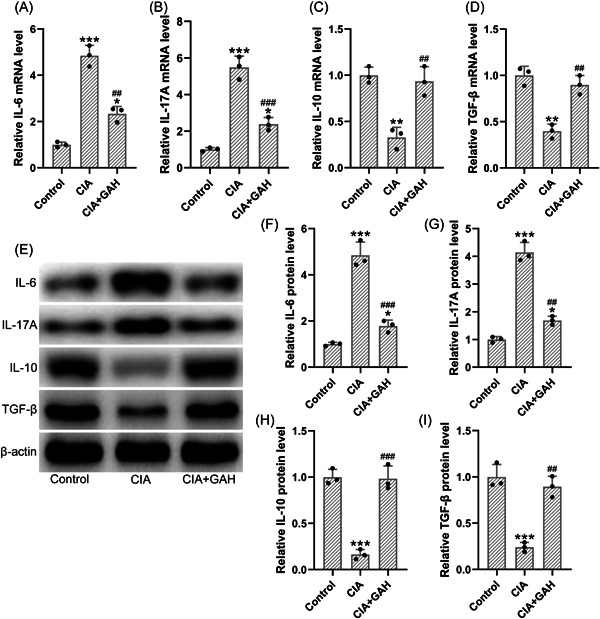
Treatment of GA‐regulated Th17/Treg‐related cytokines in the ankle joints of the mouse model of arthritis induced by collagen. The mRNA levels of IL‐6 (A), IL‐17A (B), IL‐10 (C), and TGF‐β (D) in the ankle joints were determined by using qPCR. (E) The protein expressions of those cytokines were determined by using Western blot analysis. (F–I) The relative expressions were normalized to the internal control (*n* = 3; eight tissue homogenates were mixed). *p < .05, **p < .01, and ***p < .001 as compared to the control. ^##^ *p* < .01 and ^###^ *p* < .001 as compared to the CIA group. GAH corresponded to the GA administration dosage of 80 mg/kg/d.

Finally, we explored the effects of GA on the expressions of Th17/Treg‐related cytokines in the CIA mouse model. qPCR analysis revealed an elevation of mRNA levels for the IL‐17 (Figure 5A) and IL‐6 (Figure 5B) and a reduction of mRNA levels for the IL‐10 (Figure 5C) and TGF‐β (Figure 5D). However, treatment of GA (80 mg/kg) significantly reduced the mRNA levels for the IL‐17 (Figure 5A) and IL‐6 (Figure 5B) as well as boosted the mRNA levels for the IL‐10 (Figure 5C) and TGF‐β (Figure 5D). Consistently, the treatment of GA (80 mg/kg) also suppressed the protein expressions of IL‐17 (Figure 5E,G) and IL‐6 (Figure 5F,G) and enhanced the protein expressions of IL‐10 (Figure 5E,H) and TGF‐β (Figure 5E,I). These results suggested that GA regulated the cytokines relevant to Th17/Treg balance in the CIA mouse model.

## 4 DISCUSSION

The current study explored the effects of GA on the CIA mouse model by the evaluation of RA symptoms, the expression of MMPs and inflammation‐related cytokines, the populations of Th17/Treg cells, and their cytokines. Interestingly, our results revealed that GA attenuated RA symptoms, including swelling, erythema, and loss of function of paws. Besides, GA exerted therapeutic effects for CIA mouse model in part by suppressing both local and systematic inflammation as well as restoring the Th17/Treg balance. These results suggested that GA might be effective for the treatment of RA.

CIA mouse model is a commonly used murine animal model to investigate the pathogenesis of RA and evaluate agents with therapeutic potential against RA.^22^ CIA mouse model is characterized by the infiltration of immune cells in the joint tissues including macrophages, neutrophils, and T cells, the release of cytokines and MMPs, as well as the shift in T cell subsets.^22^ Consistently, our results showed that MMPs and cytokines were significantly elevated in the ankle joint after collagen induction. Besides, the populations of Th17 cells and the levels of IL‐17 were elevated, whereas the populations of Treg cells and the levels of IL‐10 and TGF‐β were reduced in the CIA mouse model, indicating the success of the CIA model in our study.

GA is a polyphenolic compound isolated from fruits, vegetables, and herbal medicines and is well‐known for its antioxidative stress and anti‐inflammation properties.^18^, ^23^ Many studies revealed that GA exhibits broad pharmacological activities against inflammation‐related diseases including inflammatory bowel diseases, pelvic inflammation, and RA.^23^, ^24^ In 2013, Yoon et al. reported that GA exerts therapeutic potential against RA by inducing cell apoptosis and suppressing inflammatory cytokines in fibroblast‐like synoviocytes.^20^ Herein, our study is to confirm the in vivo therapeutic effects of GA on RA as well as explore the underlying mechanisms. Our results suggested that treatment of GA reduced the overexpression of MMPs and regulated the dysregulation of inflammation‐related cytokines in the CIA mouse model. These results are consistent with the previous studies, where GA exhibits inhibitory effects against inflammation cytokines and MMP family proteins.

Both Th17 and Treg cells are known to have important roles in the RA, as supported by Th17/Treg imbalance observed in preclinical and clinical studies of RA.^14^, ^25^ Th17 cells are known to mediate bone destruction and promote joint inflammation in part by secreting IL‐17.^26^ On the other hand, Treg cells suppress pro‐inflammatory responses by inhibiting the activation of T cell subsets including Th1 and Th17.^25^ As expected, we observed that the populations of Th17 cells were elevated whereas the populations of Treg cells were reduced in the CIA mouse model. These characteristics are in agreement with the previous findings, where a shift in the Th17/Treg cell to the Th17 cell side leads to RA with more severe symptoms.^9^, ^14^ Interestingly, GA reduced the populations of Th17 cells, and increased the populations of Treg cells, indicating that its therapeutic effects against RA are in part by regulating the balance between Th17 and Treg cells.

A series of cytokines, including ILs, IFN‐γ, TNF‐α, and TGF‐β, are involved in the development of RA by regulating the balance between pro‐inflammatory and anti‐inflammatory responses.^27^, ^28^ Dysregulation of cytokines is closely linked to immune cell activation.^27^ For instance, IL‐1 and TNF‐α can trigger the activation of macrophages, whereas IL‐10 can suppress Th1 cell activities by inhibiting IFN‐γ.^28^, ^29^ IL‐17 is known to induce the production of IL‐1, the latter is known as the secondary mediator for induction of arthritic changes.^30^ CIA mouse model showed an increase in the inflammation cytokines (IFN‐γ, TNF‐α, IL‐6, and IL‐17) and a decrease in the immunomodulatory cytokines (IL‐10 and TGF‐β). Besides, a shift in the Th17/Treg cell to the Th17 cell side also induces the increase of IL‐17 and the decrease of IL‐10 and TGF‐β.^11^ Interestingly, treatment of GA showed immunomodulatory effects, as supported by a decrease in the inflammation cytokines and an increase of anti‐inflammatory cytokines. In addition, the treatment of GA restored the balance between Th17 and Treg cells as well as enhanced the levels of IL‐10 and TGF‐β, and suppressed the levels of IL‐17. These results suggested that GA exerted therapeutic potential against RA by regulating the immune response.

## 5 CONCLUSION

GA exerts beneficial effects on the CIA mouse model. Treatment of GA reduced the overexpression of MMPs and regulated the dysregulation of inflammation‐related cytokines. In addition, the treatment of GA also regulated the populations of Th17/Treg cells as well as cytokines in relevant to Th17 and Treg cells. These results suggested that GA might be a good candidate for CIA therapy.

## AUTHOR CONTRIBUTIONS

**Siqin Liu**: Conceptualization (supporting); data curation (equal); writing—original draft (supporting); writing— review and editing (supporting). **Jiajia Li**: Conceptualization (supporting); data curation (equal). **Le‐heng Feng**: Conceptualization (lead); investigation (lead); methodology (lead); writing—original draft (lead); writing—review and editing (lead).

## CONFLICT OF INTEREST STATEMENT

The authors declare no conflicts of interest.

## ETHICS STATEMENT

The animal studies were approved by the First Hospital of Qiqihar. This study was performed in strict accordance with the NIH guidelines for the care and use of laboratory animals (8th edition, NIH).

## Data Availability

The data sets used and/or analyzed during the current study are available from the corresponding author upon reasonable request.
